# Intracanalicular meningioma: diagnostic by immunohistochemistry

**DOI:** 10.1016/S1808-8694(15)30769-2

**Published:** 2015-10-19

**Authors:** Andrei Borin, Daniel Mochida Okada, Oswaldo Laércio Mendonça Cruz

**Affiliations:** 1 Master, doctoral student; 2 Specialist in Otorhinolaryngology, UNIFESP/EPM; medical doctor; 3 Livre-docente habilitation, FMUSP; affiliated professor, UNIFESP/EPM. Sao Paulo Federal University

**Keywords:** immunohistochemistry, meningioma, ear

## INTRODUCTION

The meningioma (ME) was originally described by Virchow in 1863 as a tumor originating from meningothelial cells that are usually found within leptomeninges and the choroid plexi of the ventricles, with a preference for the supratentorial area.^1^ Any area that is covered by the leptomeninges, however, is potentially a site of origin for meningiomas. The first report of an exclusively intracanalicular (IC) ME has been attributed to Singh et al. in 1975.^2^ Since then, about 15 ICME have been described in the English medical literature.^1^^,^^3^

## CASE STUDY

MJRC, age 51 years, female, white, presented constant tinnitus for six months and left hypoacusis for four months. The otorhinolaryngological and neurological exams were within normal limits, except for unilateral left hearing loss (PTA – 27.5dB; discrimination – 60%). Magnetic resonance imaging showed a tumor in the internal acoustic canal, an image suggesting a vestibular schwannoma (VS). Surgery done through a middle fossa approach revealed a tumor that was redder and more adhered than usual, measuring about 10×5mm. It was located atypically in the internal acoustic canal – between the nerves – rather than in the classical posterior position.

Facial paralysis House-Brackmann grade IV presented postoperatively.

Histology reported a tumor compatible with meningothelial meningioma, which was confirmed by immunohistochemistry (S-100 – negative; EMA – positive). Audiometry was done 15 days later, demonstrating that hearing was preserved (PTA – 62.5dB). The patient recovered from facial paralysis, which had decreased to grade II six months postoperatively.

## DISCUSSION

Differentiating MEs from VSs may be difficult when MEs are exclusively IC. Both tumors affect similar age groups (45–55 yrs) and predominate in females. They also present with similar signs and symptoms, such as hearing loss and tinnitus.^1^ Facial paralysis may occur in up to 27% of ICME cases; it is, however, less common in ICVSs (about 3%).^1^^,^^3^ Radiological differentiation between both tumors is generally not possible.^1^^,^^3^

ICME surgery has certain peculiarities. Compared to ICVSs, ICMEs tend to adhere more and to be more vascularized; they may also occupy various portions of the internal acoustic canal.^1^ Such lack of predictability in the location of ICME and its relation with the VII and VIII cranial nerves may significantly increase the difficulty of surgery; the facial nerve may be displaced by the tumor to any of the quadrants in the internal acoustic canal, increasing the possibility of iatrogenic injuries.^1^^,^^3^ We defended and demonstrated the possibility of preserving postoperative hearing, as defined by the “Committee on Hearing and Equilibrium of the American Academy of Otolaryngology – Head & Neck Surgery”.4 The real possibility of preserving hearing, however, is still uncertain in ICME cases, given the paucity of case reports.

Immunohistochemistry is useful in differentiating these tumors. MEs may express both epithelial and mesenchymal markers, reflecting their double embryological origin or mesenchymal cell totipotentiality. Many markers have been used, although there is wide variation of results in the literature, which may be credited to differences in methodology.^5^ The “epithelial membrane antigen” (EMA) is generally strongly positive in MEs (84%) and negative or weakly positive and with a focal pattern in VSs. Protein S-100 is not a specific marker for neuroectodermal tissue; it may be positive in 28% of MEs.^5^ Vimentin is positive in about 95% of MEs.^5^ Table 1 shows the main immunohistochemical findings in posterior fossa tumors, based on studies by Winek^6^ and Radley.^5^ Electron microscopic ultra-structural studies should be reserved for difficult cases not clarified by immunohistochemistry, given the high cost and the technical difficulties of this method.

**Table 1:** Main immunoreactive features for differentiating posterior fossa tumors.

TUMOR	Vimentin	EMA	keratin	S-100 protein	GFAP
Meningioma	+	+	+/− (a)	+/− (b)	–
Schwannoma	+	+/− (c)	–	+	–
glioma	+	–	–	+	+
carcinoma	–	+	+	+/−	–
melanoma	+	–	–	+	–
cordoma	+	+	+	+	–

Key: EMA – epithelial membrane antigen; GFAP – glial fibrillary acidic protein.

(a) - positive in secretory meningiomas

(b) - positive in 15%

(c) - in general weak and focal when positive
